# Dietary γ-Aminobutyric Acid Promotes Growth and Immune System Performance and Improves Erythropoiesis and Angiogenesis in Gibel Carp (*Carassius auratus gibelio*)

**DOI:** 10.3390/ani15020125

**Published:** 2025-01-07

**Authors:** Xinlan Bai, Lu Zhang, Hualiang Liang, Dongyu Huang, Mingchun Ren, Haifeng Mi

**Affiliations:** 1 Wuxi Fisheries College, Nanjing Agricultural University, Wuxi 214081, China; 2 Tongwei Agricultural Development Co., Ltd., Key Laboratory of Nutrition and Healthy Culture of Aquatic, Livestock and Poultry, Ministry of Agriculture and Rural Affairs, Healthy Aquaculture Key Laboratory of Sichuan Province, Chengdu 610093, China; 3 Key Laboratory of Integrated Rice-Fish Farming Ecology, Ministry of Agriculture and Rural Affairs, Freshwater Fisheries Research Center, Chinese Academy of Fishery Sciences, Wuxi 214081, China; huangdongyu@ffrc.cn

**Keywords:** GABA, Gibel carp (*Carassius auratus gibelio*), growth performance, immune, oxygen carrying

## Abstract

This experiment aimed to investigate the effect of dietary supplementation of γ-aminobutyric acid (GABA) on the growth performance, immune response, and oxygen-transport-related factors of Gibel carp (*Carassius auratus gibelio*). An eight-week culturing experiment was designed with five experimental diets, with the actual GABA content being 368 mg/kg (G1, control group), 449 mg/kg (G2), 527 mg/kg (G3), 602 mg/kg (G4), and 675 mg/kg (G5). The results showed that the level of 527 mg/kg (G3) of GABA significantly increased the specific growth rate (SGR), weight gain rate (WGR), and final body weight (FBW) of Gibel carp, while the levels of alanine aminotransferase (ALT), aspartate aminotransferase (AST), total cholesterol (TC), and glucose (GLU) were also increased significantly. In addition, 527 mg/kg (G3) and 602 mg/kg (G4) of GABA significantly increased the total antioxidant capacity (T-AOC). The mRNA expression of *tnf-α*, *tgf-β*, and *il-10* was significantly increased at the level of 449 mg/kg (G2). In terms of oxygen-carrying capacity, the mRNA expression of *epo*, *tf*, *tfr1*, *ho-1*, and *vegf* was markedly increased at the level of 449 mg/kg (G2). In conclusion, dietary GABA supplementation can boost growth performance, enhance the immune system, and increase oxygen-carrying capacity in Gibel carp.

## Simple Summary

This study explored the effects of γ-aminobutyric acid (GABA) on growth, immunity, erythropoiesis, and angiogenesis in Gibel carp (*Carassius auratus gibelio*). Gibel carp were fed with five levels of GABA for a total of eight weeks. This study showed that the growth performance of Gibel carp was improved, the immune performance was enhanced, and the expression of erythropoietic and angiogenic genes was also enhanced. In conclusion, GABA had a positive effect on the growth of Gibel carp.

## 1. Introduction

With the increasing emphasis on healthier diets, aquatic-animal-derived foods such as pelagic fish and salmon have become widely sought after due to their considerably higher nutrient content compared to cattle and sheep [1]. The increased global demand for blue food has led to greater challenges for the aquaculture industry [2]. In 2022, aquaculture accounted for 59% of global fishery production [3], with members of the *Cyprinidae* family being the main species produced globally, accounting for 18% of total aquatic animal production [3]. With the increasing demand, the aquaculture industry has undergone a shift from coarse to intensive farming [4], and the degree of aquaculture intensification has increased. However, this intensification has been accompanied by a greater susceptibility of fish to the occurrence and spread of disease [5]. One effective way to enhance disease resistance and improve the immunity of fish is the application of feed additives [4].

As a non-protein amino acid, γ-aminobutyric acid (GABA) is widely found in both plants and animals, being first discovered in potato tubers by Steward in 1949 [6]. GABA can promote the growth of plants and animals. In plants, GABA participates in the plant life cycle [7] and has a positive regulatory effect on plant growth and yield [8,9]. In livestock and poultry, GABA has been shown to promote the growth of broiler chickens by significantly increasing their feed intake (FI) and body weight (BW), as well as reducing their feed conversion ratio (FCR) [10]. It has also been shown to promote the growth of beef cattle [11] and pigs [12] through an increase in average daily gain (ADG) and a decrease in FCR. In aquatic animals, dietary GABA improves the growth performance of Nile tilapia (*Oreochromis niloticus*) [13,14] and Pacific oysters (*Crassostrea gigas*) [15]. Similar results have been found in pharaoh cuttlefish (*Sepia pharaonis*) [16], Jian carp (*Cyprinus carpio var. Jian*) [17], whiteleg shrimp (*Litopenaeus vannamei*) [18,19], juvenile olive flounder (*Paralichthys olivaceus*) [20], and grass carp (*Ctenopharyngodon idellus*) [21].

GABA is also involved in the immune system regulation of both plants and animals. GABA helps plants to cope with environmental stresses and effectively increases their antioxidant properties [22,23]. In livestock and poultry, the addition of exogenous GABA alleviated the stress of broiler chickens caused by high-density farming [24] and attenuated the oxidative stress and inflammatory response induced by dexamethasone (DEX) [10]. Similarly, GABA decreased the expression of pro-inflammatory factors and increased the activity of antioxidant enzymes in pigs [25]. In aquatic animals, GABA has been shown to serve an essential function within the immune system of sticklebacks (*Gasterosteus aculeatus*) [26]. GABA also enhances the immune response and antioxidant status of Nile tilapia [13,14] and maintains immune homeostasis, while inhibiting phagocytosis and the apoptosis of immune cells in Pacific oysters [15]. The dietary addition of GABA has been demonstrated to mitigate oxidative stress in pharaoh cuttlefish [16]. Furthermore, dietary GABA has been shown to lessen tissue damage and attenuate inflammatory responses in largemouth bass (*Micropterus salmoides*), thereby enhancing its antioxidant capacity [27]. Additionally, dietary GABA serves to modulate the inflammatory response of Chinese mitten crabs (*Eriocheir sinensis*) by diminishing detrimental metabolites [28]. GABA also helps organisms to increase their oxygen-carrying capacity [29,30,31,32]. Studies have shown that GABA is inextricably linked to the generation of vascular networks [33,34] and also has direct vasodilatory effects [35]. For example, with the help of GABA, mrigal carp (*Cirrhinus mrigala*) [29] and crucian carp (*Carassius carassius*) [36] increased their oxygen-carrying capacity through vasodilation. However, current research on GABA mainly focuses on immunity and antioxidant aspects [13,21,37], with less research on its effects on oxygen-carrying capacity. Therefore, it is urgently required to carry out relevant research to investigate the impact of GABA supplementation on the oxygen-carrying capacity of Gibel carp, while also exploring its role in the growth and immunity of Gibel carp.

Plasma status is closely linked to the health of fish. Plasma biochemical indicators, such as alanine aminotransferase (ALT) and aspartate aminotransferase (AST), are released into the plasma when liver tissue is damaged and are considered liver injury markers [38]. However, they also play a role in nutrient metabolism [39]. In fish, superoxide dismutase (SOD) and catalase (CAT) are considered the main antioxidant enzymes [40]. The level of malondialdehyde (MDA) is an indicator of the degree of oxidative stress damage. And increased MDA levels can cause cell damage and produce biological toxicity [41]. Total antioxidant capacity (T-AOC) serves as an index to assess all antioxidant capabilities, including enzymatic and nonenzymatic antioxidant activities [42]. Hypoxia-inducible transcription factor 1 (HIF-1) is one of the most important regulatory factors in the body’s response to hypoxia; it maintains the normal function of cells by regulating metabolism and blood vessels in hypoxic environments [43]. It regulates erythropoiesis and angiogenesis by controlling downstream target genes, thereby helping the body to enhance its oxygen-carrying capacity.

Gibel carp (*Carassius auratus*), an important economic fish species in China, accounts for about 10% of freshwater fishery resources [44]. However, environmental stresses such as global weather changes [45,46], intensive aquaculture [4], and drug abuse [47,48], which lead to growth inhibition, have been shown to reduce immunity and affect the respiratory system and oxygen-carrying capacity in Gibel carp [49]. These stresses result in major economic losses. In addition, with the shift to intensive farming, Gibel carp subjected to the consequent stressful conditions have reduced organismal immunity. As such, these fish are more likely to die when afflicted by disease, which results in yield loss [4]. Although the important functions of GABA [50] and its precursors glutamate [51,52] and glutamine [53,54,55] within animal immune systems are well known, they have been relatively little studied in Gibel carp. Therefore, this study aimed to investigate the effect of adding dietary GABA on the growth, antioxidant properties, immune response, and oxygen-carrying capacity of Gibel carp.

## 2. Materials and Methods

### 2.1. Diet Preparation

This study divided fish into five groups. The feed formula is listed in Table 1. The formulations had the same ingredients except for the different GABA contents. The first to fifth groups corresponded to the additional GABA levels of 0 mg/kg (G1, control group), 90 mg/kg (G2), 180 mg/kg (G3), 270 mg/kg (G4), and 360 mg/kg (G5), respectively. The composition of the GABA content in each experimental diet was evaluated and analyzed using high-performance liquid chromatography (HPLC, Sykam, Free State of Bavaria, Eresing, Germany) following the procedure of Lee et al. [56]. The measured value of each group was 368 mg/kg (G1), 449 mg/kg (G2), 527 mg/kg (G3), 602 mg/kg (G4), and 675 mg/kg (G5), respectively. The ingredients were mixed with the help of water and oil according to Table 1 and were pelletized on a feeder (F-26(II), South China University of Technology, Guangzhou, China). After drying the feed, we placed them in proper self-sealing bags and then stored them at −20 °C for later use.

### 2.2. Experimental Management

The current study was conducted at the Wuxi Fisheries College of Nanjing Agricultural University (Wuxi, China). To achieve domestication prior to experimentation, Gibel carp were staged in net cages (1 m × 1 m × 1 m) for 14 days. With three repetitions in each group, three hundred fish with a mean weight of 41.87 ± 0.04 g were taken and randomly divided into 15 floating cages (1 m × 1 m × 1 m) and kept for eight weeks. Throughout the farming process, Gibel carp were fed to satiation until they stopped swimming to the surface to feed, with feedings conducted at 8 a.m. and 4 p.m. The temperature range was 30 ± 2 °C, the dissolved oxygen level was kept above 6.0 mg/L, the pH level was within 7.0 to 7.8, and total ammonia nitrogen was below 0.1 mg/L throughout the feeding process.

### 2.3. Sample Collection

Samples were collected after a 24 h fasting period at the end of the 8-week aquaculture experiment. All Gibel carp were weighed for each cage. With the use of MS-222 (100 mg/L), nine fish from each group (three fish from each replicate) were selected randomly after anesthesia, and blood was collected, with the plasma being obtained via centrifugation (3500× *g*, 10 min, 4 °C). These fish were then harvested for intestinal tissue to analyze the intestinal antioxidant indices. Finally, two fish from each cage, selected according to the principle of randomness, were stored at −20 °C for the determination of whole body composition. All samples were stored in a freezer at −80 °C for further analysis.

### 2.4. Chemical Analysis

Using the method of AOAC (2003) [57], the crude protein, ash, and crude lipid content of the feed and whole body composition were measured. In brief, we first placed the sample in a glass container and dried it in an oven at 105 °C to obtain the moisture content. Then, we crushed the sample into a powder and stored it in a self-sealing bag for subsequent use. We measured the crude protein content of the dried sample with a Haineng K1100 instrument (Jinan Haineng Instrument Co., Ltd., Jinan, China) using the Kjeldahl method. We analyzed the crude lipid content of the powdered sample using the Soxhlet extraction method on an automatic analyzer (Haineng SOX606, Jinan Haineng Instrument Co., Ltd., Jinan, China) and obtained the ash data by calcining the sample in a muffle furnace (XL-2A, Hangzhou Zhuochi Instrument Co., Ltd., Hangzhou, China) at 560 °C for 6 h. The indices of plasma biochemical analysis were evaluated using a Mindray BS-400 analyzer (Shenzhen, China), a method also mentioned in our previous studies [39]. Indices were measured using the machine and corresponding Mindray kits in accordance with the manufacturer’s protocols. The detection indices were as follows: alanine aminotransferase (ALT), albumin (ALB), aspartate aminotransferase (AST), total cholesterol (TC), total protein (TP), triglycerides (TG), and glucose (GLU).

Indicators of intestinal antioxidant capacity, such as SOD and CAT, and the contents of nonenzymes, such as MDA and T-AOC, were measured using commercial kits from Nanjing Jiancheng Bioengineering Institute (Nanjing, China), following the instructions provided in the reagent manuals. See our previous study for specific methods [58].

### 2.5. Gene Expression Analysis

The primers used are listed in Table 2. Through the use of RNA extraction reagents (Vazyme, Nanjing, China), RNA was extracted from the liver tissue of the Gibel carp. We then quantified RNA. A PCR system was activated and operated as per the predetermined settings. The procedure for a One-Step SYBR Prime Script TM PLUS RT-PCR kit (Takara, Dalian, China) is as follows: reverse transcription for 5 min at 42 °C, pre-denaturation for 10 s at 95 °C, and then denaturation at 95 °C for 5 s and 60 °C for 30 s, 40 cycles. Stable β-actin was used as a nonregulated reference gene, as previously used in a Gibel carp study [58], and no significant changes were found in this study or the previous one. The mRNA levels were calculated based on the standard curve method [59].

### 2.6. Data Analysis

The data were subjected to normality and homogeneity tests. Using SPSS (22.0), data were analyzed via one-way ANOVA and Tukey’s test for pairwise comparison (*p* < 0.05). The statistics are shown as the mean ± standard error.

## 3. Results

### 3.1. Growth Performance

Table 3 indicates the growth performance of the Gibel carp. As shown in the table, the level of 527 mg/kg (G3) of GABA significantly increased the specific growth rate (SGR), weight gain rate (WGR), and final body weight (FBW) (*p* < 0.05) of the fish compared to the control group. It also helped to reduce the FCR (*p* > 0.05). However, the FCR was increased by the level of 675 mg/kg (G5) (*p* > 0.05).

### 3.2. Whole Body Composition

Table 4 indicates the body composition of the Gibel carp. Regarding the four indices, no significant difference between each component and the control group was found (*p* > 0.05). The crude lipid content was elevated by the level of 602 mg/kg (G4) of GABA, but there was no significant difference compared with the control group (*p* > 0.05).

### 3.3. Plasma Biochemistry

Table 5 shows the plasma biochemistry results. When GABA was added at a level of 602 mg/kg (G4), the levels of ALB, TP, and TG were significantly reduced compared to the control group (*p* < 0.05). When GABA was added at a level of 527 mg/kg (G3), the levels of AST, ALT, TC, and GLU were increased significantly compared to the control group (*p* < 0.05). When the GABA level was 602 mg/kg (G4), the level of TC decreased significantly compared to the control group (*p* < 0.05).

### 3.4. Plasma Enzymatic Indices

Table 6 presents the plasma antioxidant indices. It can be seen that the contents of T-AOC were significantly increased at the levels of 527 mg/kg(G3) and 602 mg/kg (G4) mg/kg (*p* < 0.05). For SOD, CAT, and MDA, there was no significant difference between each group and the control group (*p* > 0.05). However, SOD and CAT had better vitality at the level of 449 mg/kg (G2) (*p* > 0.05). At the level of 675 mg/kg (G5), the level of MDA showed the lowest value (*p* > 0.05).

### 3.5. Nrf2 Signaling Pathway

**Figure 1:**
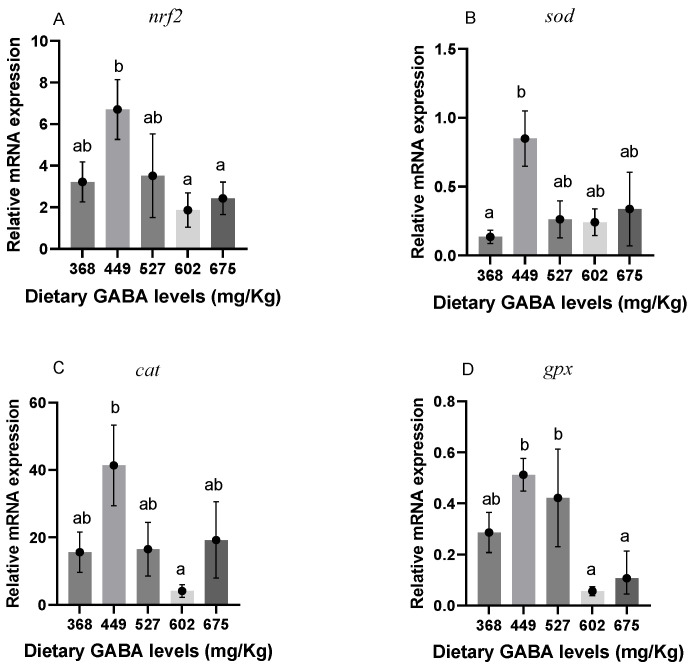
Genes related to *Nrf2* signaling pathway in Gibel carp. ^ab^ Different letters represent significant differences between groups and no letter represents no significant difference between groups. (**A**) *nrf2*; (**B**) *sod*; (**C**) *cat*; and (**D**) *gpx*.

The gene expression of *Nrf2* signaling pathway-related factors is shown in Figure 1. As Figure 1B shows, *sod* was significantly increased with the GABA level of 449 mg/kg (G2) (*p* < 0.05). However, as shown in Figure 1A,C,D, *nrf2*, *cat*, and *gpx* did not significantly differ from the control group (*p* > 0.05).

### 3.6. Liver Inflammatory Factor Genes

**Figure 2:**
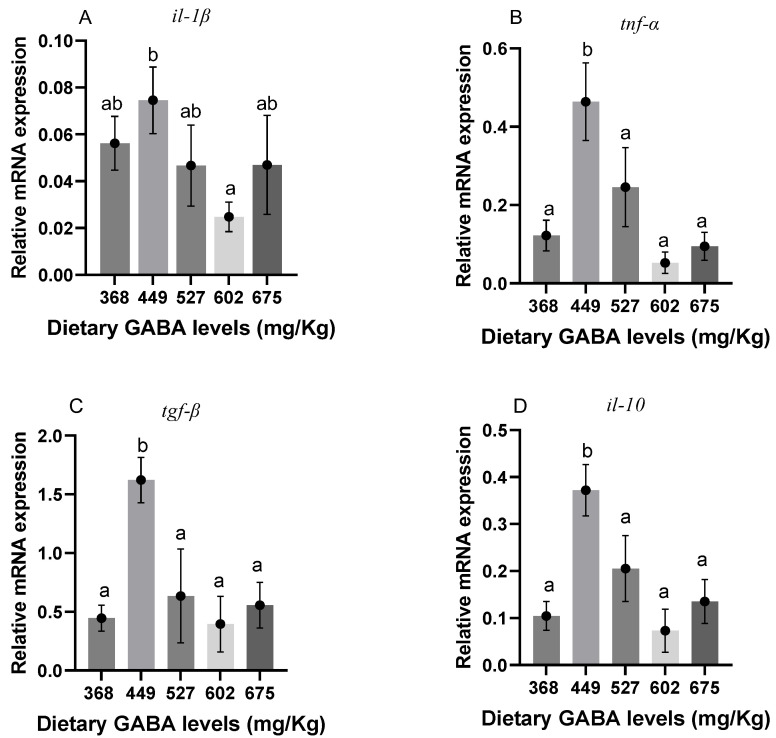
Genes related to inflammatory factors in liver of Gibel carp. ^ab^ Different letters represent significant differences between groups and no letter represents no significant difference between groups. (**A**) *il-1β*; (**B**) *tnf-α*; (**C**) *tgf-β*; and (**D**) *il-10*.

Figure 2 illustrates the expression levels of pro-inflammatory factors. There was no significant difference between the additive and control group in the expression of *il-1β*, as shown in Figure 2A (*p* > 0.05). As Figure 2B shows, the mRNA expression of *tnf*-α was increased significantly at 449 mg/kg (G2) of GABA (*p* < 0.05). As shown in Figure 2C,D, *tgf-β* and *il-10* were significantly increased at the level of 449 mg/kg (G2) (*p* < 0.05).

### 3.7. Genes Related to Vascular Regeneration and Iron Metabolism

**Figure 3:**
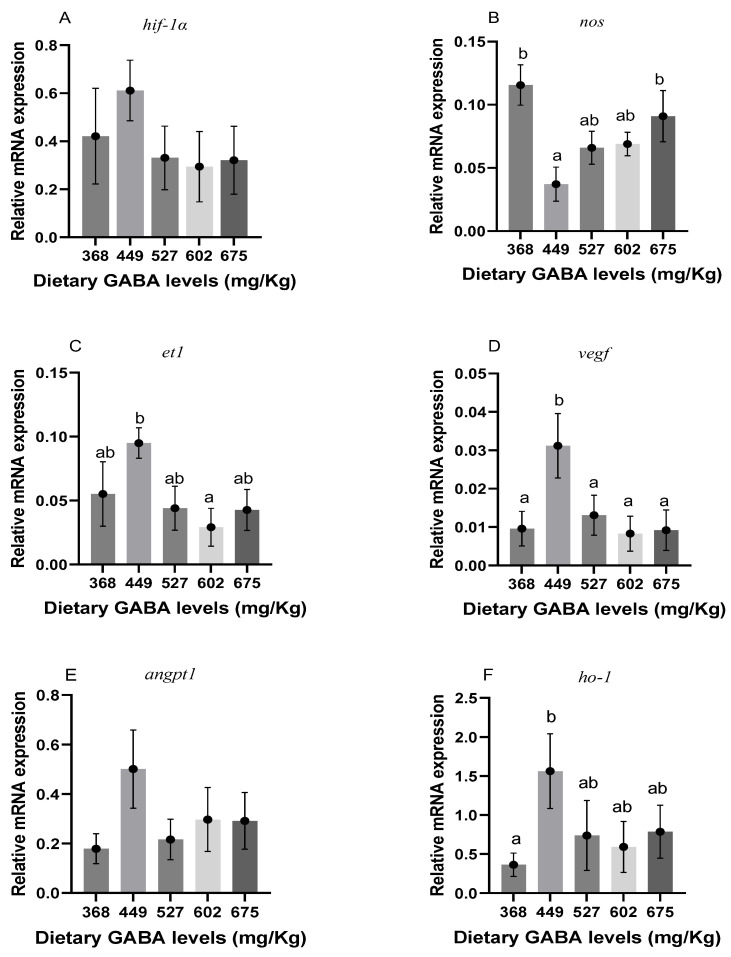
Genes related to vascular regeneration-related genes in Gibel carp. ^ab^ Different letters represent significant differences between groups and no letter represents no significant difference between groups. (**A**) *hif-1α*; (**B**) *nos*; (**C**) *et1*, endothelin 1; (**D**) *vegf*; (**E**) *angpt1*; and (**F**) *ho-1*.

**Figure 4:**
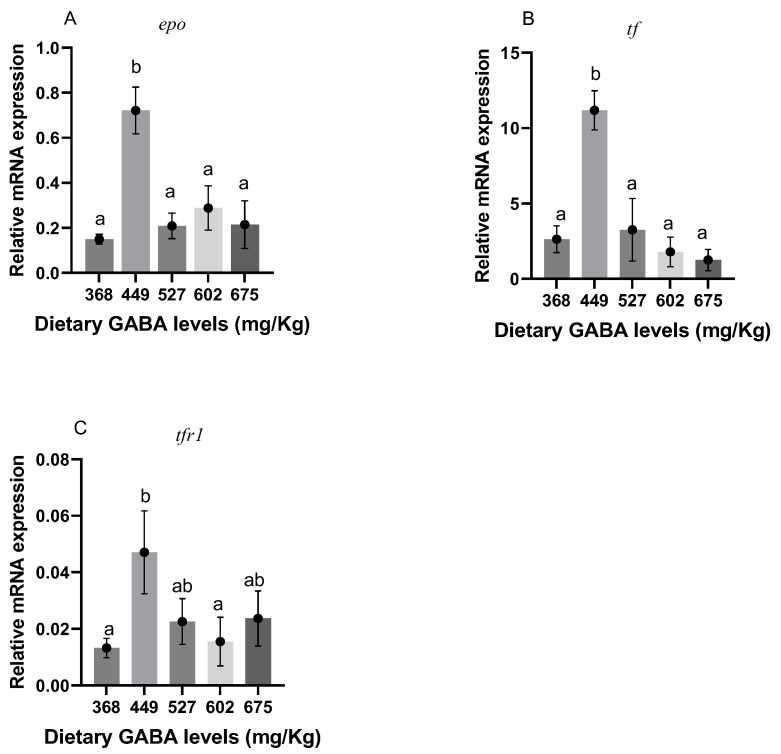
Genes related to iron metabolism in Gibel carp. ^ab^ Different letters represent significant differences between groups and no letter represents no significant difference between groups. (**A**) *epo*; (**B**) *tf*; and (**C**) *tfr1*.

In Figure 3C–F, it can be seen that the expression of *et1*, *vegf*, *angpt1*, and *ho-1* peaked at the level of 449 mg/kg (G2) (*p* < 0.05). *Ho-1* and *vegf* were increased significantly at this level (*p* < 0.05). As shown in Figure 3B, the expression of *nos* was significantly decreased at 449 mg/kg (G2) of GABA (*p* < 0.05). As shown in Figure 3A, the expression of *hif-1* in each group showed no significant difference compared to the control group (*p* > 0.05). The expression of genes related to iron metabolism in fish can be seen in Figure 4. As shown in Figure 4A–C, *epo*, *tf*, and *tfr1* were all significantly increased at the level of 449 mg/kg (G2) of GABA, and the gene expression peaked at this level (*p* < 0.05).

## 4. Discussion

In this study, it was found that the level of 527 mg/kg (G3) of GABA significantly improved the growth performance of Gibel carp to a certain extent. However, excess GABA did not promote the growth of Gibel carp but rather inhibited it. Similar results were found in whiteleg shrimp [18,19], where dietary supplementation with GABA promoted growth to a certain extent, but the growth performance did not consistently improve with an increase in the added amounts. Similarly, a moderate supplementation of dietary GABA also enhanced the growth performance of the Chinese mitten crab [55]. As such, the addition of GABA did not show a dose/effect response, which indicates that there was a suitable range of GABA addition, and excessive GABA supplementation might reduce growth performance. This is most likely caused by excessive GABA affecting the Ca^2+^ levels in Gibel carp pituitary cells, leading to an inhibition of the release of growth hormones [56]. In this study, GABA was effective in promoting the growth of Gibel carp when added at 527 mg/kg (G3). Interestingly, according to the quadratic regression analysis of FE, the optimal level of GABA in pharaoh cuttlefish [16] and Jian carp [17] was 55.3 and 96.75 mg/kg, respectively, perhaps due to the differences in species and size. This finding also favors GABA as a dietary additive in fish feeds. In relation to whole fish body composition, the addition of GABA increased the crude protein content of Gibel carp to some extent; however, the difference was not significant. Similar outcomes were found in grass carp [21] and whiteleg shrimp [18]. For glutamine, the precursor of GABA had a significant effect on the crude protein content of gilthead seabream (*Sparus aurata*) [57] and Jian carp [61]. The enhancement of protein retention in fish bodies may be due to the added dietary GABA being broken down after ingestion by the organism, thereby improving the nutritional metabolism of the Gibel carp liver. This might also explain the level of crude protein content of Gibel carp in this study.

The central and integrative role of plasma in fish physiology means that plasma status can generally reflect fish health [18,62,63]. In the present study, the level of 527 mg/kg (G3) GABA significantly increased levels of AST, ALT, and TC. The addition of GABA also increased the level of AST and ALT in crucian carp (*Carassius carassius*) [37]. By contrast, higher doses of GABA significantly reduced the viability of ALT and AST in Chinese mitten crab larvae [64]. ALT and AST were also involved in nutrient metabolism [39]. Thus, it can be concluded that the elevation of ALT and AST levels in this study was due to the vigorous nutrient metabolism in the fish rather than tissue injury. A similar conclusion was found in largemouth bass [65]. The significantly higher GLU of all the groups that added different levels of GABA also supports this view at some levels. In addition, TP and TG showed a fluctuating trend with an initial rise followed by a decline, with a significant decrease at 602 mg/kg (G4), which is further evidence of vigorous metabolism. This has also been observed in largemouth bass [65].

As an intermediate product of oxygen metabolism, excessive ROS leads to oxidative stress in the organism [66]. In response to oxidative stress, antioxidant-related molecules such as SOD and CAT come into effect [67]. In previous studies, the antioxidant capacity of grass carp was enhanced by the addition of both glutamate—a precursor of GABA [68]—and dietary amino butyric acid [21]. Similar results were found not only in grass carp but also in whiteleg shrimp [19], Chinese mitten crab [64], and even in mammals [69]. However, in this study, the levels of MDA, as well as the activities of CAT and SOD, did not show a significant correlation with the level of GABA addition. This difference might be due to the different species, specification sizes, and the culture environment. Similar to this study, feeding GABA did not significantly alter the SOD of olive flounder [70]. However, it was found that T-AOC showed a significant enhancement in this study at GABA additions of 527 mg/kg (G3) and 602 mg/kg (G4). Similarly, by enhancing T-AOC activity, GABA also significantly improved the antioxidant capacity in turbot (*Scophthalmus maximus* L.) [71] and growing minks (*Neovison vison*) [72]. In addition, the GABA level of 449 mg/kg (G2) upregulated the mRNA expression of *nrf2*, which, in turn, increased the expression of downstream *cat*, *sod*, and *gpx*, as well as *ho-1*-related genes. Among them, *sod* and *ho-1* were also significantly increased at the level of 449 mg/kg (G2), which indicated that GABA has the ability to mobilize antioxidant enzymes to assist the Gibel carp in resisting oxidative stress. This is why *sod* increased significantly, whereas SOD did not, suggesting that it is likely that transcriptional regulation of genes alters the process of gene expression [73]. Accordingly, a certain amount of GABA might help the organism alleviate the damage caused by oxidative stress and improve the activity of antioxidant enzymes in coping with oxidative stress.

Inflammation is a self-defense response when an organism is subjected to internal or external stimuli. Studies have demonstrated that GABA lowers the generation of inflammatory cytokines through the activation of specific receptors [74,75]. *Tnf-α* is a key driver of immune responses [76], but in fish, *tnf-α* exhibits the complexity of cytokine-regulated immunity [77]. *Tnf-α* acts as an activator and mediator of phagocytosis in rainbow trout (*Oncorhynchus mykiss*) [78] and goldfish (*Carassius auratus* L.) macrophages [79], which suggests that pro-inflammatory factors also contribute substantially to the body’s fight against infection. In the context of this study, *tnf-α* was significantly increased at the level of 449 mg/kg (G2). Moreover, *tnf-α* can enhance the resistance to infection by increasing the killing power of macrophages and stimulating the immune system. The results of this experiment revealed that the level of GABA did not significantly affect the mRNA expression of *il-1β*. Meanwhile, the mRNA expressions of *il-10* and *tgf-β*, as factors playing a role in fighting inflammation, demonstrated a similar trend to the mRNA expression of *tnf-α*, with significantly higher expression at the level of 449 mg/kg (G2). The mRNA expression of *il-10* and *tgf-β* has been shown to serve a regulatory function in fish immunity [80,81]. Dietary GABA enhanced immunity in Jian carp by significantly increasing the mRNA expression of *il-10* [17], and GABA-induced elevation expression of *tnf-α* and *tgf-β* was also present in Nile tilapia [82], which is similar to our experimental results. The significant increase in its expression at this dosage occurred because the levels of 449 mg/kg (G2) made Gibel carp more sensitive to infection and made it easier for them to mobilize the body to respond to the stimulus. However, it did not cause tissue damage. Hence, the significant increase in *tnf-α* at 449 mg/kg (G2) might play a role in stimulating the immune system, which in turn mobilizes factors *tgf-β* and *il-10* to respond positively against the impending inflammation, thus improving the body’s resistance to infection and immunity.

There are many ways for an organism to improve its oxygen-carrying capacity [83]. In a previous study, exogenous GABA supplementation increased the oxygen-carrying capacity of mrigal carp and contributed to a reduction in the upregulation of *hif-1* [29], which mirrors the results of the present study. Downstream *hif-1α*, *et1*, *vegf*, *angpt1*, *epo*, *tf*, and *tfr1* factors showed the same trend as *hif-1α*, except *nos*. The elevated mRNA expression of *angpt1* and the significant increase in mRNA of *vegf* when GABA was added at the level of 449 mg/kg (G2) also indicated the promotional effect of GABA on the angiogenesis of Gibel carp. Similar conclusions could also be drawn in relation to mice [84]. In terms of vascular tone, the mRNA expression of *et1* showed an opposite trend to the mRNA expression of *nos*. The level of 449 mg/kg (G2) of GABA promoted the mRNA expression of *et1* and increased the vascular tone of Gibel carp, whereas the mRNA expression of *nos* was significantly reduced at the level of 449 mg/kg (G2). Unlike endothelial GABA [85], exogenous GABA showed a tendency to inhibit the mRNA expression of *nos*, which might be due to the inhibition by *tgf-β* [86]. In addition, the level of 449 mg/kg (G2) of GABA significantly promoted the mRNA expression of *epo*, *tf*, and *tfr1*. This also indicated that GABA can promote the production of erythrocytes, iron transport proteins, and their receptors in Gibel carp, as well as improve the oxygen-carrying capacity of the organism.

## 5. Conclusions

Our experimental results suggest that 527 mg/kg (G3) of GABA can significantly improve the growth performance and metabolism of Gibel carp and that 449 mg/kg (G2) of GABA can enhance immunity by modulating inflammatory factors and achieve optimal levels of iron metabolism and angiogenesis factors. In summary, our research indicates that the optimal amount of GABA to promote the growth of Gibel carp is 527 mg/kg (G3), and the optimal amount to enhance the immunity and oxygen-carrying capacity of Gibel carp is 449 mg/kg (G2). This study provides basic guidelines for the practical application of GABA supplementation and feeding strategies.

## Figures and Tables

**Table 1:** Ingredient and nutrient composition of the experimental feed (% air dry basis).

Ingredients			
Fish meal ^a^	14.00	Calcium dihydrogen phosphate	2.00
Poultry meal ^a^	4.00	Vitamin premix ^b^	0.20
Soybean meal ^a^	22.00	Mineral premix ^b^	2.00
Cottonseed meal ^a^	5.00	98.5%L-Lysine ^c^	0.30
Rapeseed meal ^a^	22.00	DL-Methionine ^c^	0.10
Wheat flour ^a^	14.15	Vc Phospholipids	0.05
Rice bran ^a^	10.00− ^d^	60%Choline chloride	0.20
Soybean oil	4.00	γ-Aminobutyric acid (mg/kg)	^d^
Analyzed proximate composition			
Crude protein (%)		40.25 ± 0.28	
Crude lipid (%)		8.16 ± 0.11	

^a^ Fish meal, obtained from Wuxi Tongwei Feedstuffs Co., Ltd., Wuxi, China; soybean meal, crude protein 46.0%, crude lipid 4.3%, obtained from Wuxi Tongwei Feedstuffs Co., Ltd., Wuxi, China; cottonseed meal 53.7%, crude lipid 1.4%; rapeseed meal, crude protein 39.2%, crude lipid 6.1%, obtained from Wuxi Tongwei Feedstuffs Co., Ltd., Wuxi, China; wheat flour, crude protein 13.1%, crude lipid 4.0%, obtained from Wuxi Tongwei Feedstuffs Co., Ltd., Wuxi, China. ^b^ Vitamin premix (IU or mg/kg of premix). Mineral premix (g/kg of premix). Both of them were obtained from HANOVE Animal Health Products (Wuxi, China). ^c^ L-lysine and DL-Methionine obtained from Feeer Co., Ltd. (Shanghai, China). ^d^ Means additional GABA levels (0 mg/kg (G1), 90 mg/kg (G2), 180 mg/kg (G3), 270 mg/kg (G4), and 360 mg/kg (G5), respectively) of five diets.

**Table 2:** Primer sequences.

Genes ^a^	Forward Primer (5′-3′)	Reverse Primer (5′-3′)	Accession Number/Reference
*β-actin*	TCCATTGTTGGACGACCCAG	TGGGCCTCATCTCCCACATA	LC382464.1
*gpx*	GAAGTGAACGGTGTGAACGC	GATCCCCCATCAAGGACACG	DQ983598.1
*nrf2*	TACCAAAGACAAGCAGAAGAAACG	GCCTCGTTGAGCTGGTGTTTGG	[60]
*cat*	TGAAGTTCTACACCGATGAG	CTGAGAGTGGACGAAGGA	XM_026238665.1
*sod*	TCGGAGACCTTGGTAATGT	CGCCTTCTCATGGATCAC	JQ776518.1
*il-10*	AGTGAGACTGAAGGAGCTCCG	TGGCAGAATGGTGTCCAAGTA	[61]
*il-1β*	GCGCTGCTCAACTTCATCTTG	GTGACACATTAAGCGGCTTCA C	[62]
*tgf-β*	GTTGGCGTAATAACCAGAAGG	AACAGAACAAGTTTGTACCGATAAG	[62]
*tnf-α*	CATTCCTACGGATGGCATTTACTT	CCTCAGGAATGTCAGTCTTGCAT	[62]
*hif-1α*	CTGCCGATCAGTCTGTCTCC	TTTGTGGAGTCTGGACCACG	DQ306727.1
*vegf*	ATCGAGCACACGTACATCCC	CCTTTGGCCTGCATTCACAC	NM_131408.3
*nos*	GGGGACCCTCCTGAAAATGG	TTCTGTCCTCAACGCTGGTG	AY644726.1
*et1*	TAAAGCAGCGTCAGACAGGG	CTGCCAGCTTGTGTTTGCAT	NM_131519.1
*angpt1*	CCAAACCTCACCAAGCAAGC	GGATTACAGTCCAGCCTCCG	XM_059556208.1
*ho-1*	GCAAACCAAGAGAAGCCACC	GGAAGTAGACGGGCTGAACC	KC758864
*epo*	CGAAGTGTCAGCATACCGGA	GCAGATGACGCACTTTTCCC	KC460317.1
*tf*	CCGAGAAGATGCACGCAAAG	TGTGCATGCCTTGACCAGAT	AF518747.1
*tfr1*	CTTTGTCAACGAAGTGGCTGAAT	TACCAAAGAAAATGTGGCGGAAC	XM_052542523.1

^a^ *β-actin*, beta-actin; *gpx*, glutathione peroxidase; *nrf2*, nuclear factor erythroid 2-related factor 2; *cat*, catalase; *sod*, superoxide dismutase; *il-10*, interleukin-10; *il-1β*, interleukin-1β; *tgf-β*, transforming growth factor-β; *tnf-α*, tumor necrosis factor-α; *hif-1α*, hypoxia inducible factor-1α; *vegf*, vascular endothelial growth factor; *nos*, nitric oxide synthase; *et1*,endothelin-1; *angpt1*, angiopoietin-1; *ho-1*, heme oxygenase 1; *epo*, erythropoietin; *tf*, transferrin; *tfr1*, transferrin receptor protein 1.

**Table 3:** Growth performance.

Indices	G1	G2	G3	G4	G5
IBW (g)	41.83 ± 0.04	41.88 ± 0.06	41.82 ± 0.03	41.93 ± 0.04	41.90 ± 0.05
FBW (g)	100.68 ± 0.87 ^a^	103.33 ± 2.20 ^ab^	107.40 ± 1.23 ^b^	102.97 ± 2.42 ^ab^	98.67 ± 1.69 ^a^
FCR	1.30 ± 0.02 ^ab^	1.26 ± 0.05 ^ab^	1.18 ± 0.02 ^a^	1.27 ± 0.05 ^ab^	1.35 ± 0.04 ^b^
WGR (%)	140.68 ± 2.33 ^a^	146.73 ± 5.58 ^ab^	156.83 ± 2.86 ^b^	145.56 ± 5.97 ^ab^	135.49 ± 4.15 ^a^
SGR (%/d)	0.94 ± 0.01 ^a^	0.97 ± 0.02 ^ab^	1.01 ± 0.01 ^b^	0.97 ± 0.03 ^ab^	0.92 ± 0.02 ^a^

Note: IBW (g)—sum of initial body weight of each cage/number of each cage in the beginning; FBW (g)—sum of final body weight of each cage/number of each cage at the end; FCR—weight of feed consumed during feeding/weight gain of fish (g); SGR (%/d)—(ln fish final average weight (g) − ln fish initial average weight (g))/(days of culture) × 100; WGR (%)—(final body average weight of fish (g) − initial body average weight (g))/initial body weight (g) × 100. Data are mean values of triplicate. ^ab^ Different superscripts mean significant difference (*p* < 0.05).

**Table 4:** Whole body composition.

Indices	G1	G2	G3	G4	G5
Moisture (%)	76.62 ± 0.15	75.83 ± 0.52	75.93 ± 0.39	75.96 ± 0.51	76.12 ± 0.51
Protein (%)	15.38 ± 0.27	15.89 ± 0.48	16.28 ± 0.70	16.29 ± 0.72	16.36 ± 0.51
Lipid (%)	2.19 ± 0.35	1.93 ± 0.52	2.12 ± 0.63	2.43 ± 0.37	1.81 ± 0.41
Ash (%)	4.50 ± 0.13	4.64 ± 0.29	4.57 ± 0.12	4.52 ± 0.08	4.96 ± 0.24

Note: Data are mean values of triplicate measurements.

**Table 5:** Plasma biochemistry.

Indices	G1	G2	G3	G4	G5
ALB (g/L)	9.19 ± 0.28 ^bc^	9.62 ± 0.37 ^c^	9.48 ± 0.32 ^bc^	7.71 ± 0.22 ^a^	8.64 ± 0.17 ^b^
ALT (U/L)	0.81 ± 0.16 ^a^	1.11 ± 0.13 ^ab^	1.58 ± 0.32 ^b^	1.48 ± 0.21 ^ab^	1.27 ± 0.19 ^ab^
AST (U/L)	134.54 ± 4.56 ^a^	149.75 ± 9.27 ^ab^	159.50 ± 8.54 ^b^	143.00 ± 9.55 ^ab^	142.93 ± 4.61 ^ab^
TC (mmol/L)	5.76 ± 0.22 ^b^	6.34 ± 0.28 ^b^	6.02 ± 0.26 ^b^	4.99 ± 0.19 ^a^	5.93 ± 0.24 ^b^
TG (mmol/L)	1.21 ± 0.05 ^b^	1.47 ± 0.09 ^b^	1.28 ± 0.07 ^b^	1.14 ± 0.06 ^a^	1.43 ± 0.07 ^b^
GLU (mmol/L)	5.29 ± 0.47 ^a^	7.11 ± 0.36 ^b^	7.13 ± 0.33 ^b^	7.52 ± 0.38 ^b^	7.76 ± 0.22 ^b^
TP (g/L)	28.77 ± 0.82 ^b^	30.61 ± 1.32 ^b^	29.08 ± 1.11 ^b^	25.56 ± 0.32 ^a^	27.88 ± 0.76 ^b^

Note: Data are mean values of triplicate measurements. ^abc^ Different superscripts mean significant difference (*p* < 0.05).

**Table 6:** Plasma antioxidant indices.

Indices	G1	G2	G3	G4	G5
SOD (U/mL)	19.39 ± 0.32	20.05 ± 0.55	19.88 ± 0.58	19.69 ± 0.86	18.47 ± 0.75
MDA (nmol/mL)	10.63 ± 1.02	10.02 ± 0.71	9.04 ± 0.48	10.04 ± 0.82	8.78 ± 0.63
CAT (U/mL)	12.11 ± 3.02	14.97 ± 2.51	13.72 ± 3.14	9.02 ± 1.51	7.92 ± 1.32
T-AOC (mM)	0.32 ± 0.007 ^a^	0.33 ± 0.005 ^a^	0.35 ± 0.004 ^b^	0.34 ± 0.003 ^b^	0.33 ± 0.005 ^ab^

Note: Data are mean values of triplicate measurements. ^ab^ Different superscripts mean significant difference (*p* < 0.05).

## Data Availability

Data are contained within the article.
